# Higher vascular endothelial growth factor-C concentration in plasma is associated with increased forearm capillary filtration capacity in breast cancer-related lymphedema

**DOI:** 10.14814/phy2.12403

**Published:** 2015-06-09

**Authors:** Mads Radmer Jensen, Lene Simonsen, Tonny Karlsmark, Charlotte Lanng, Jens Bülow

**Affiliations:** 1 Department of Clinical Physiology and Nuclear Medicine, Bispebjerg Hospital, University Hospital of CopenhagenCopenhagen, Denmark; 2 Department of Dermatology, Copenhagen Wound Healing Centre, Copenhagen Lymphoedema Centre, Bispebjerg Hospital, University Hospital of CopenhagenCopenhagen, Denmark; 3 Department of Breast Surgery, Herlev Hospital, University Hospital of CopenhagenCopenhagen, Denmark; 4 Department of Biomedical Sciences, University of CopenhagenCopenhagen, Denmark

**Keywords:** Breast cancer, capillary filtration coefficient, cytokine, inflammation, lymphedema, suction blister, VEGF-C, venous occlusion plethysmography

## Abstract

Breast cancer-related lymphedema (BCRL) is a frequent, chronic and debilitating swelling that mainly affects the ipsilateral arm and develops as a complication to breast cancer treatment. The pathophysiology is elusive opposing development of means for prediction and treatment. We have earlier shown that the forearm capillary filtration coefficient (CFC) is increased bilaterally in BCRL. In this study, we aimed to elucidate if increased CFC is associated with low-grade inflammation and/or vascular endothelial growth factor-c (VEGF-C) signaling. Fourteen patients with unilateral BCRL and nine matched breast cancer controls without BCRL participated. Forearm CFC was measured by venous congestion strain gauge plethysmography, and suction blisters were induced medially on the upper arms. Concentrations of 17 selected cytokines, VEGF-C, and total protein were measured in blister fluid and in plasma. Forearm CFC was higher bilaterally in BCRL subjects (*P* ≤ 0.036). No differences between forearms were found in either group. Plasma VEGF-C concentrations were significantly higher in the BCRL subjects (*P* < 0.001). In BCRL subjects, monocyte chemotactic protein 1 (MCP-1) (*P* = 0.009) and total protein (*P* = 0.035) concentrations were higher in blister fluid from edematous arms compared with nonedematous arms. No differences were found in interstitial cytokine or total protein concentrations between arms in control subjects. Higher plasma concentration of VEGF-C is a possible cause of bilaterally increased forearm CFC in BCRL subjects. Interstitially increased MCP-1 levels may augment local microvascular protein permeability in BCRL.

## Introduction

Breast cancer is the most frequent cancer in women worldwide. Breast cancer-related lymphedema (BCRL) – a chronic progressive swelling that mainly affects the ipsilateral upper extremity – is a common and debilitating complication to breast cancer treatment (Gartner et al. 2010). Although several factors, e.g., axillary lymph node dissection and obesity have been shown to increase the risk of BCRL development (DiSipio et al. 2013), the pathophysiology of BCRL remains poorly understood opposing development of means for prediction and treatment. In later stages of lymphedema, tissue remodeling results in increased subcutaneous adipose tissue volume, fibrosis, and skin thickening (Brorson et al. 2009; Lin et al. 2012). The classical model of lymphedema pathophysiology, namely that lymphedema is principally an edema high in protein content resulting from insufficient lymphatic drainage (Mortimer 1998), fails to explain this remodeling. Furthermore, we have recently shown that the forearm capillary filtration coefficient (CFC), i.e., microvascular fluid filtration capacity is increased in BCRL. Interestingly, CFC was also larger in the contralateral nonedematous forearm in BCRL subjects compared with healthy age-matched controls (Jensen et al. 2013). High CFC may be *a constitutional biological trait of the patients*, which theoretically will predispose to BCRL development due to a relatively smaller reserve lymphatic drainage capacity in relation to the high CFC; it may be caused by BCRL per se; or it may be an effect of breast cancer and/or its treatment itself. The aim of this study was to test these hypotheses with specific focus on the possibility that bilaterally increased forearm CFC found in BCRL subjects is an effect of mediators developing concomitantly with the lymphedema. Both chronic low-grade inflammation (Avraham et al. 2013; Lin et al. 2012) and vascular endothelial growth factor C (VEGF-C) signaling (Joory et al. 2003; Miaskowski et al. 2013) have been associated with BCRL, and both can cause increased microvascular permeability. In lymphedema, the interstitial sojourn time of intermediate and high-molecular weight mediators that affect microvascular permeability is likely increased due to reduced interstitial clearance (Miller et al. 2011). A secondary effect thereof may be increased microvascular permeability both to water (hydraulic conductivity) and to proteins (Reed and Rubin 2010). To elucidate this, we measured forearm capillary filtration coefficients bilaterally and quantified total protein, selected cytokines, and VEGF-C in plasma and in tissue fluid obtained by the suction blister technique in women with BRCL and in matched breast cancer patient controls, who had not developed BCRL >2 years after surgery.

## Methods

### Subjects

This study was approved by *The Committees on Health Research Ethics in the Capital Region of Denmark* (protocol number H-2-2012-137). All subjects gave written informed consent prior to participation. Common inclusion criteria were as follows: Treatment for unilateral invasive breast carcinoma with axillary dissection (ALND) and adjuvant radiation and chemotherapy (including taxanes) according to Danish breast cancer treatment guidelines; otherwise healthy; age 35–65 years; no history of erysipelas and no antihypertensive and/or anti-inflammatory medical treatment. Specific inclusion criteria for the BCRL group were: >6 months since last breast cancer treatment; clinical signs of lymphedema, e.g., swelling, edema, and skin thickening; and in addition ≥5% increased volume of the edematous upper extremity compared with the contralateral (Dylke et al. 2012). Specific control group inclusion criteria were: >2 years since breast cancer surgery; no symptoms or clinical signs of BCRL; and a relative arm volume difference less than 4% regardless of arm dominance. The groups were matched with regard to age, body mass index (BMI, kg/m^2^), anti-estrogen treatment, and time since breast cancer surgery.

### Objective measures of lymphedema

BCRL was objectively quantified by the measurements of upper extremity volumes and local total skin water content using opto-electric perometry (Perometer 1000M®, Pero-System Messgeräte GmbH, Wuppertahl, Germany) and Tissue Dielectric Constant (TDC) measurements (MoistureMeter D Compact^,^ Delfin Technologies Ltd, Kuopio, Finland) as described previously (Jensen et al. 2013). Briefly, upper extremity volume was measured from the knuckles of the hand to the anterior axillary fold with the arm extended and abducted to 90°. TDC – a physical quantity without entity that is directly proportional to total tissue water – was measured locally on the site of maximum clinical signs of BCRL in an effective depth of 2.5 mm, and on the corresponding site on the contralateral arm. This was usually on the volar forearm. In the control group, TDC was measured on the volar forearm 10 cm distal to the cubital fossa. Visible veins were avoided.

### Capillary filtration coefficient

The capillary filtration coefficient (CFC) is a measure of the fluid filtration capacity of the microcirculation and is dependent on the microvascular permeability to water (hydraulic conductivity) and surface area available for filtration (Starling 1896; Gamble et al. 1993; Levick and Michel 2010). Forearm CFC was calculated by linear regression of forearm capillary filtration rates at step-wise increases in venous congestion pressures measured bilaterally and simultaneously by venous congestion strain gauge plethysmography using a programmable plethysmograph with electrical strain gauge calibration (AI6®; D.E. Hokanson, Inc., Bellevue, WA) as described previously (Jensen et al. 2013). Briefly, BCRL patients agreed to pause compression sleeve treatment for at least 12 h prior to measurements to achieve a relatively stable arm volume. Examinations were conducted in a quiet temperature controlled laboratory (22–24^°^C) in the morning. Subjects acclimatized for 30 min of which the last 15 min were supine rest in order to achieve haemodynamic steady state. Pressure cuffs were placed around the upper arms and mercury-in-rubber strain gauges around the largest circumference of the forearms. The length of each strain gauge was individually selected to ensure skin contact with least possible tension to reduce pitting of the strain gauge in the skin during prolonged venous congestion. Venous congestion pressure steps were 35, 50, and 65 mmHg. The duration of the pressure steps was gradually increased by 20 sec with each step increments in cuff pressure starting at 3 min and ending at 3 min and 40 sec. This design aimed to compensate for the positive relation between venous distension time (initial nonlinear volume increase after venous congestion) and cuff pressure ensuring sufficient linear curve segments for reliable measurement of the filtration rate at higher cuff pressures (Gamble et al. 1993) while keeping the venous congestion period relatively short. Relative forearm volume change in relation to time was recorded continuously. Forearm capillary filtration rates for each congestion pressure were calculated off-line (AI6 software, D.E. Hokanson Inc.) as the slope of linear curve segments with minimum duration of 60 sec. The investigator was blinded to subject grouping and operated side. The CFC (*μ*L/100 g/mmHg/min) was calculated by linear regression of the measured capillary filtration rates and venous congestion pressures (cuff pressure).

### Plasma samples

Venous blood was collected in 9-mL EDTA tubes from a cubital vein in the arm contralateral to the operated side. Plasma was separated by centrifugation at 4500 rpm for 8 min at 4°C and immediately frozen at -30°C until analysis.

### Suction blister samples

Lymphedema patients are prone to the development of erysipelas. Therefore, in order to minimize the invasiveness of interstitial fluid sampling skin suction blister fluid was chosen as a surrogate for interstitial fluid for measurements of concentrations of total protein, VEGF-C and cytokines (Haaverstad et al. 1996; Davidsson et al. 2013). Since blister induction may cause local skin hyperpigmentation, we induced blisters on the medial upper arm (5 cm proximal to the elbow). Clinically evident lymphedema at this site was therefore mandatory in the BCRL group, although this was not always the site of maximum swelling. Custom suction cups were manufactured from 35 × 25 × 8 mm Plexiglas plates. A 6-mm deep hole with a diameter of 8 mm was bored in the center, and connected to a metal nuzzle via a 1 mm diameter suction channel bored from one plate end. Disinfected suction cups were attached bilaterally to the skin surface with tape (TransporeTM; 3M Healthcare, Copenhagen, Denmark). Prior, the skin was carefully disinfected with alcohol wipes. Both suction cups were connected to the same electric vacuum pump using 2-mm inner diameter silicone tubing in a parallel system. A negative pressure of -200 mmHg was applied for 90 min. To further promote blister formation, the suction cups and surrounding skin was heated to 39°C using heating lamps. Skin temperature was monitored frequently to ensure constant temperature. The result is a blister filled with a straw-yellow fluid, which is delimited by the roof consisting of viable epidermis and the floor being the basement membrane (Kiistala 1968). The blister fluid was carefully and completely aspirated using a sterile disposable 27-gauge needle and a 1 mL syringe, transferred to a 0.5 mL Eppendorf tube and immediately frozen at −30°C until analysis. After, the epidermis was carefully replaced, and the area bandaged with TegadermTM Film (3M) for at least 7 days.

### Cytokine quantification

To the best of our knowledge, no reports of interstitial cytokine concentrations in BCRL have been published. We therefore decided to screen the suction blister fluid for a range of relevant cytokines. Due to limited sample volume, we chose the xMAP multiplex technology (Houser 2012) on a Luminex 100TM system (Luminex Corp., Hertogenbosch, the Netherlands) running Bio-Plex ManagerTM software. Others have shown that it is possible to quantify a range of different cytokines in suction blister fluid using this technique (Dearman et al. 2004; Janssens et al. 2009; Davidsson et al. 2013). Two identical (same LOT number) human cytokine 17-plex assays where purchased (Bio-Plex ProTM Human Magnetic Cytokine, Bio-Rad Laboratories Inc., Copenhagen, Denmark) for measurement of concentrations of interleukin-1*β* (IL-1*β*), IL-2, IL-4, IL-5, IL-6, IL-7, IL-8, IL-10, IL-12, IL-13, IL-17, Granulocyte Colony Stimulating Factor (G-CSF), Granulocyte-Monocyte Colony Stimulating Factor (GM-CSF), Interferon-*γ* (IFN-*γ*), Monocyte Chemotactic Protein-1 (MCP-1), Macrophage Inflammatory Protein-1 (MIP-1), and Tumor Necrosis Factor-*α* (TNF-*α*) in plasma and suction blister fluid samples. All samples were diluted 1:3 in diluent for plasma samples. Plasma and suction blister fluid samples from the same subject were placed on the same plate, and samples from both subject groups were distributed on both plates. Cytokine concentrations were determined in duplicates. A nine-point standard curve was generated to enable measurements of low cytokine concentrations. Measured points of the standard curve that deviated >20% from the expected concentration given by the vendor were regarded as unreliable and discarded. Only sample concentrations in range of the edited standard curve were analyzed. In a separate assay, VEGF-C was quantified using the same method and vendor. Additionally, we aimed to verify MCP-1 concentrations in suction blister fluid found using the Luminex system by the ELISA technique (ab179886 – MCP1 (CCL2) Human SimpleStep ELISATM Kit; Abcam®, Cambridge, UK). Assays were applied according to manufacturer specifications; however, in a reduced number of samples (*n* = 17) due to limited residual sample volumes.

### Total protein quantification

Total protein was quantified by means of the Bradford colorimetric method (Bradford 1976). In the applied assay, the absorption is linearly correlated with total protein concentration up to 0.5 mg/mL (Total protein kit, Micro; Sigma-Aldrich, Denmark ApS, Brøndby, Denmark). The sample dilution factor was determined based on preliminary experiments on plasma and suction blister fluid samples. A high dilution factor was necessary in order obtain absorption values in the linear range of the assay. Hence, 2 *μ*L sample was diluted 1:200 with 0.9% sodium chloride. The protein standard was human albumin 0.3 mg/mL (Sigma Aldrich) and the negative control was 0.9% sodium chloride. Samples were distributed on the plates as described above, and absorption was measured in duplicates at 590 nm using a microplate reader (Multiskan R MCC/340 reader, Labsystems, Basingstoke, UK).

### Statistics

Results are presented as median (min–max). Paired tests were used for within subject comparisons, i.e., edematous versus nonedematous arms in BCRL subjects and ipsilateral versus contralateral arms in control subjects. Unpaired tests were used for group comparisons. Normally distributed data were compared using the *t*-test. Skewed data were log-transformed. If the transformation was unsuccessful, a nonparametric test was applied on the raw data. Some cytokine concentrations were below the detection limit of the assay (out of range <, OOR<). In these cases, a maximum of 20% OOR< (*n* = 2 in BCRL subjects and *n* = 1 in controls) was allowed. If >20% OOR< the results were not analyzed. Due to multiple comparisons and the derived risk of type 1 errors, the significance level was set to *P* = 0.01, while *P* in the 0.01–0.05 range was considered a trend.

## Results

### Subjects

Fourteen patients with BCRL and nine controls completed the study protocol. No serious adverse reactions occurred, specifically no infections or worsening/elicitation of lymphedema occurred.

In the BCRL group, median time from axillary lymph node dissection to development of edema was 7 months (range, 4–50 months). Match and treatment data are shown in Table1.

**Table 1:** Match - and treatment data for the study population. Quantitative data are given as median (min–max) and treatment data in %.

	BCRL (*n* = 14)	Control (*n* = 9)	*P*
Age (years)	55 (41–65)	55 (44–64)	0.756
BMI (kg/m^2^)	29.6 (22.5–34.0)	27.4 (24.2–39.2)	0.988
No. lymph nodes excised	15 (12–30)	19 (13–36)	0.281
Time since ALND (months)	42 (22–137)	48 (39–57)	0.979
Mastectomy	50%	67%	0.669
Adjuvant chemotherapy (including Docetaxel)	100%	100%	1.000
Ongoing anti-oestrogen therapy	86%	89%	0.611

### Objective measures of edema

Upper extremity volumes in the BCRL group were 3539 (3049–4862) mL in the edematous arms compared with 3092 (2701–3947) mL in the nonedematous arms (*P* < 0.01). The excess volume defined as the edematous/nonedematous ratio was 1.15 (1.05–1.33) in the BCRL group. In the control group, upper extremity volumes were 3343 (2310–4412) mL in the ipsilateral arm and 3378 (2324–4482) mL in the contralateral (*P* = 0.607).

TDC in the BCRL group was 43 (33–58) on the maximum site of edema in the edematous arms and 27 (23–35) on the corresponding site in the contralateral arms (*P* < 0.01). In the control group, TDC was 26 (22–30) on the ipsilateral arm and 26 (23–28) on the contralateral (*P* = 0.261).

### Microvascular filtration rates and capillary filtration coefficients

**Figure 1:**
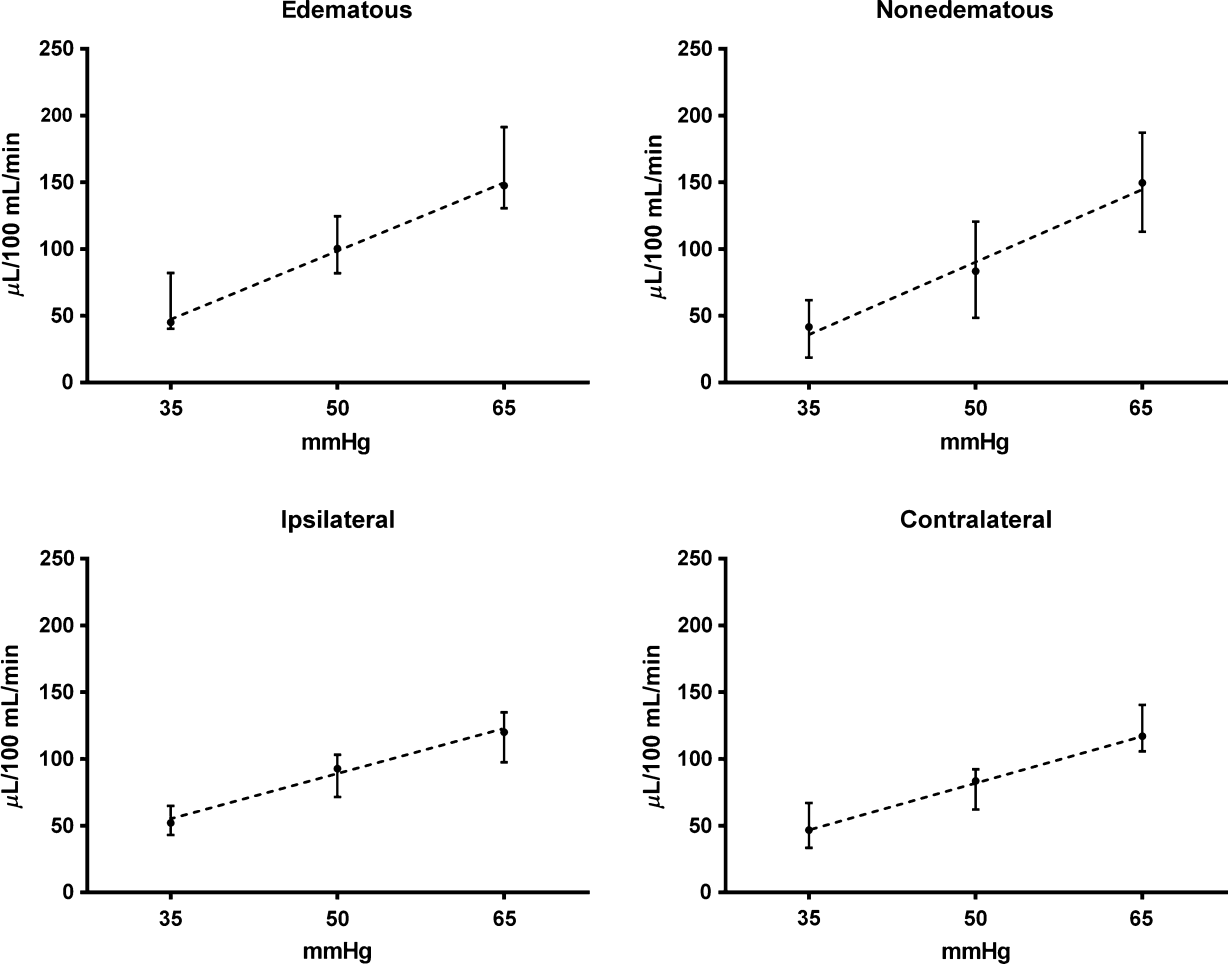
Forearm microvascular filtration rates (*μ*L/100 g/min) in the BCRL (*n* = 13) - and the control (*n* = 9) groups showing the linear relation between filtration rate and cuff pressure (mmHg). Filled dots mark medians and whiskers show interquartile range. Note the steeper slope in both the forearms in the BCRL group compared with controls.

In one subject with BCRL, filtration curves were too erratic to measure reliable filtration rates. Summed measured microvascular filtration rates in relation to cuff pressures are illustrated in Fig.1. In the BCRL group, median (min–max) CFC was 3.01 (2.14–6.21) *μ*L/100 g/mmHg/min in the edematous forearms and 3.21 (1.69–6.52) *μ*L/100 g/mmHg/min in the nonedematous forearms (*P* = 0.895). In the control group, median (min–max) CFC was 2.45 (1.51–3.99) *μ*L/100 g/mmHg/min in the ipsilateral forearms and 2.68 (1.35–3.09) *μ*L/100 g/mmHg/min in the contralateral forearms (*P* = 0.880). However, CFCs in edematous forearms in the BCRL group were larger compared with the ipsilateral forearms in the control group (*P* = 0.036). Likewise, CFCs in the nonedematous contralateral forearms in the BCRL group were larger compared with the corresponding in the control group (*P* = 0.032).

### Cytokines in plasma

**Figure 2:**
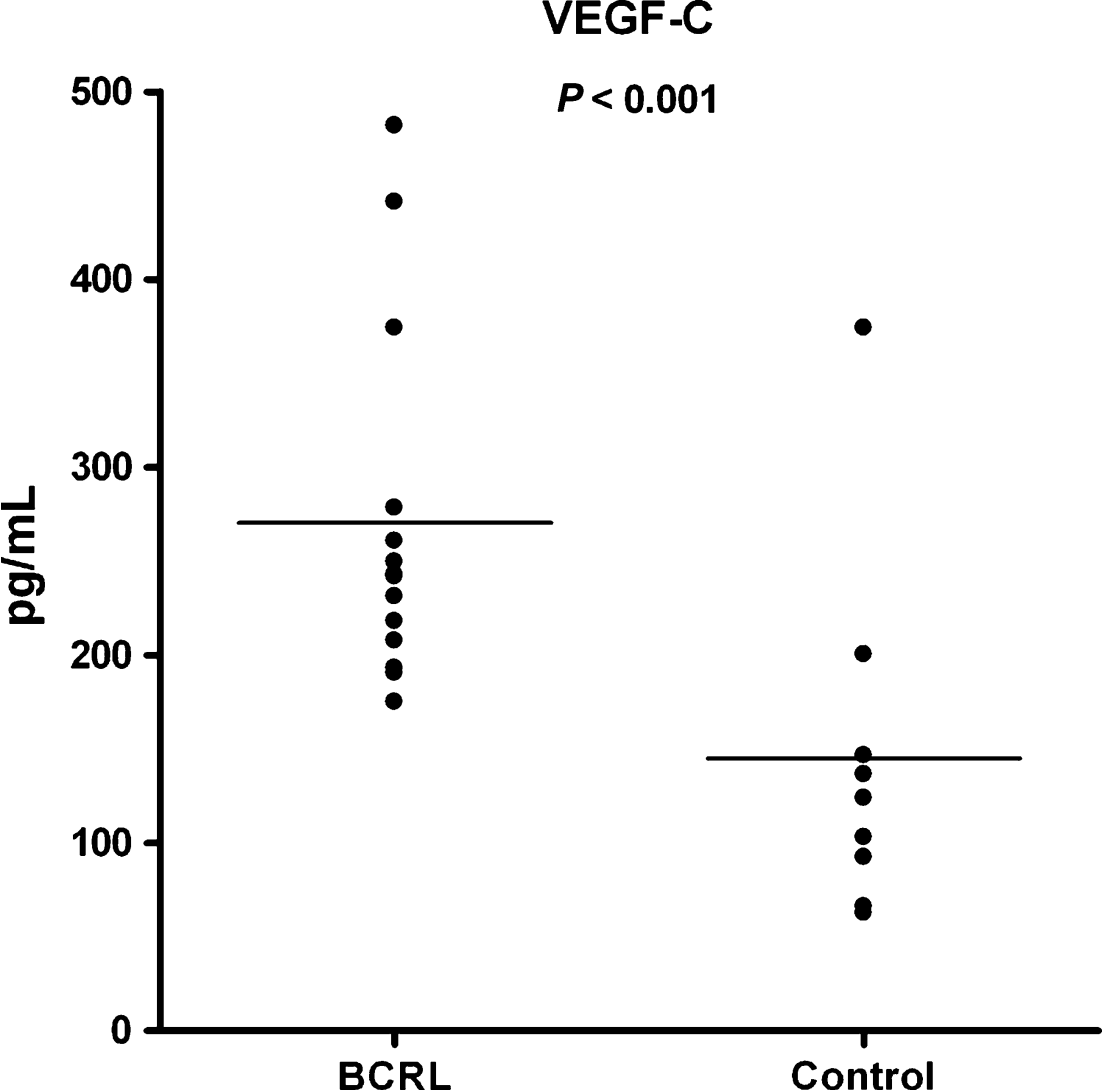
Aligned dot-plot of plasma concentrations of VEGF-C in the BCRL - (*n* = 14) and the control group (*n* = 9).

VEGF-C concentrations are depicted in Fig.2. In the BCRL group, VEGF-C was 242.4 (175.2–481.9) pg/mL compared with 124.0 (62.8–374.3) pg/mL in the control group (*P* < 0.001). Concentrations of the remaining analyzed cytokines are shown in Table2. IL-1*β*, IL-2, IL-5, IL-10, IL-17, GM-CSF and IFN-*γ* were excluded from analysis (data not shown) due to too many values out of range below the detection limit of the standard curve (OOR<). No values were above the detection limit of the standard curve for any cytokine.

**Table 2:** Concentrations of analyzed cytokines in plasma in pg/mL shown as median and range (min–max) with *n* values below the detection limit of the standard curve (OOR<). For overview *P* > 0.1 is depicted as nonsignificant (n.s.).

Cytokine	BCRL (*n* = 14)		Controls (*n* = 9)		*P*
	OOR< (*n*)	Median (min–max), pg/mL	OOR< (*n*)	Median (min–max), pg/mL	
IL-4	0	0.8 (0.2–4.3)	0	2.4 (0.3–7.9)	ns
IL-6	0	5.1 (2.7–30.3)	0	15.1 (1.8–58.2)	ns
IL-7	0	4.9 (2.7–19.0)	0	10.4 (2.2–33.8)	ns
IL-8	0	9.8 (6.4–39.4)	0	21.3 (4.7–67.8)	ns
IL-12	2	27.7 (13.9–319.3)	1	63.9 (22.8–169.4)	ns
IL-13	1	1.7 (1.0–10.4)	1	6.6 (1.5–19.4)	0.028
G-CSF	1	76.2 (26.6–199.8)	0	96.3 (42.8–166.3)	ns
MCP-1	1	55.0 (26.0–98.6)	0	65.9 (23.0–158.1)	ns
MIP-1	0	119.6 (67.3–192.6)	0	149.6 (111.4–202.4)	ns
TNF-α	0	6.8 (2.5–34.3)	1	20.1 (8.8–78.5)	0.053

### Cytokines in suction blisters fluid

Blister fluid volumes ranged from 35 *μ*L to 150 *μ*L (data not shown) with no significant difference between groups or upper extremities. Concentrations for analyzed cytokines in suction blister fluid samples are given in Table3. In BCRL subjects, MCP-1 concentrations were significantly higher in the edematous arms compared with the paired nonedematous arms (*P* = 0.009). MCP-1 concentrations in the same samples measured by ELISA (data not shown) were on average three fold higher; however; a significant correlation between the two techniques was found (*R*^2^ = 0.6, *P* < 0.001). No significant differences in any cytokine concentrations were found between the edematous arms in the BCRL group compared with the ipsilateral arms in the control group (*P* ≥ 0.161). IL-1*β*, IL-2, IL-4, IL-5, IL-12, IL-13, IL-17, G-CSF and IFN-*γ* were not analyzed (data not shown) as too many values were below the detection limit of the standard curve (OOR<). Due to insufficient volume in some blister samples after completion of the 17 cytokine and total protein assays (see below), measurements of VEGF-C concentrations were performed in a reduced number of samples. In the BCRL group, VEGF-C concentrations were 168.1 (63.1–327.0) pg/mL in the edematous arms (*n* = 9) and 134.6 (103.0–312.5) pg/mL in the nonedematous arms (*n* = 8). In the control group, VEGF-C concentrations were 87.3 (70.0–185.6) in the ipsilateral arms (*n* = 7) and 99.9 (75.3–173.6) pg/mL in the contralateral arms (*n* = 7).

**Table 3:** Concentrations of analyzed cytokines in suction blister fluid shown as median (min–max) in pg/mL with *n* values out of range values below the detection limit of the standard curve (OOR<). For overview *P* > 0.1 is depicted as nonsignificant (n.s.).

Group	Cytokine	Oedematous/Ipsilateral		Non-oedematous/Contralateral		*P*
		OOR< (*n*)	Median (min–max), pg/mL	OOR< (*n*)	Median (min–max), pg/mL	
BCRL (*n* = 14)	IL-6	1	18.5 (4.1–166.5)	0	22.7 (7.5–92.8)	n.s.
	IL-7	1	8.2 (3.0–228.8)	0	5.3 (2.2–317.7)	n.s.
	IL-8	1	41.2 (9.2–386.2)	0	86.4 (11.5–363.7)	n.s.
	IL-10	0	13.9 (6.9–336.1)	0	17.6 (5.3–363.5)	n.s.
	GM-CSF	0	388.9 (130.9–1265.1)	0	296.7 (76.0–1486.8)	n.s.
	MCP-1	1	250.9 (99.4–711.8)	0	199.8 (38.1–298.5)	0.009
	MIP-1	1	205.8 (76.1–574.8)	0	213.1 (70.7–414.0)	n.s.
	TNF-α	2	16.6 (2.1–101.2)	0	17.3 (1.6–254.0)	n.s.
Control (*n* = 9)	IL-6	0	12.7 (5.1–184.7)	1	27.6 (5.1–129.4)	n.s.
	IL-7	0	6.5 (3.8–372.1)	1	9.4 (3.7–40.5)	n.s.
	IL-8	0	55.8 (20.8–193.9)	1	68.1 (32.2–191.6)	n.s.
	IL-10	0	18.9 (6.4–53.7)	0	23.3 (7.9–45.1)	n.s.
	GM-CSF	0	434.7 (162.4–481.3)	0	356.1 (143.3–770.1)	n.s.
	MCP-1	0	228.4 (115.8–511.9)	1	233.3 (146.4–556.8)	n.s.
	MIP-1	0	241.1 (128.2–461.3)	1	277.4 (130.3–350.2)	n.s.
	TNF-α	0	17.5 (2.7–171.2)	1	28.0 (5.5–156.3)	n.s.

### Total protein

In plasma, total protein concentration was 52.7 (41.8–86.2) mg/mL in the BCRL group and 54.1 (38.3–59.4) mg/mL in the control group with no significant difference (*P* = 0.742).

**Figure 3:**
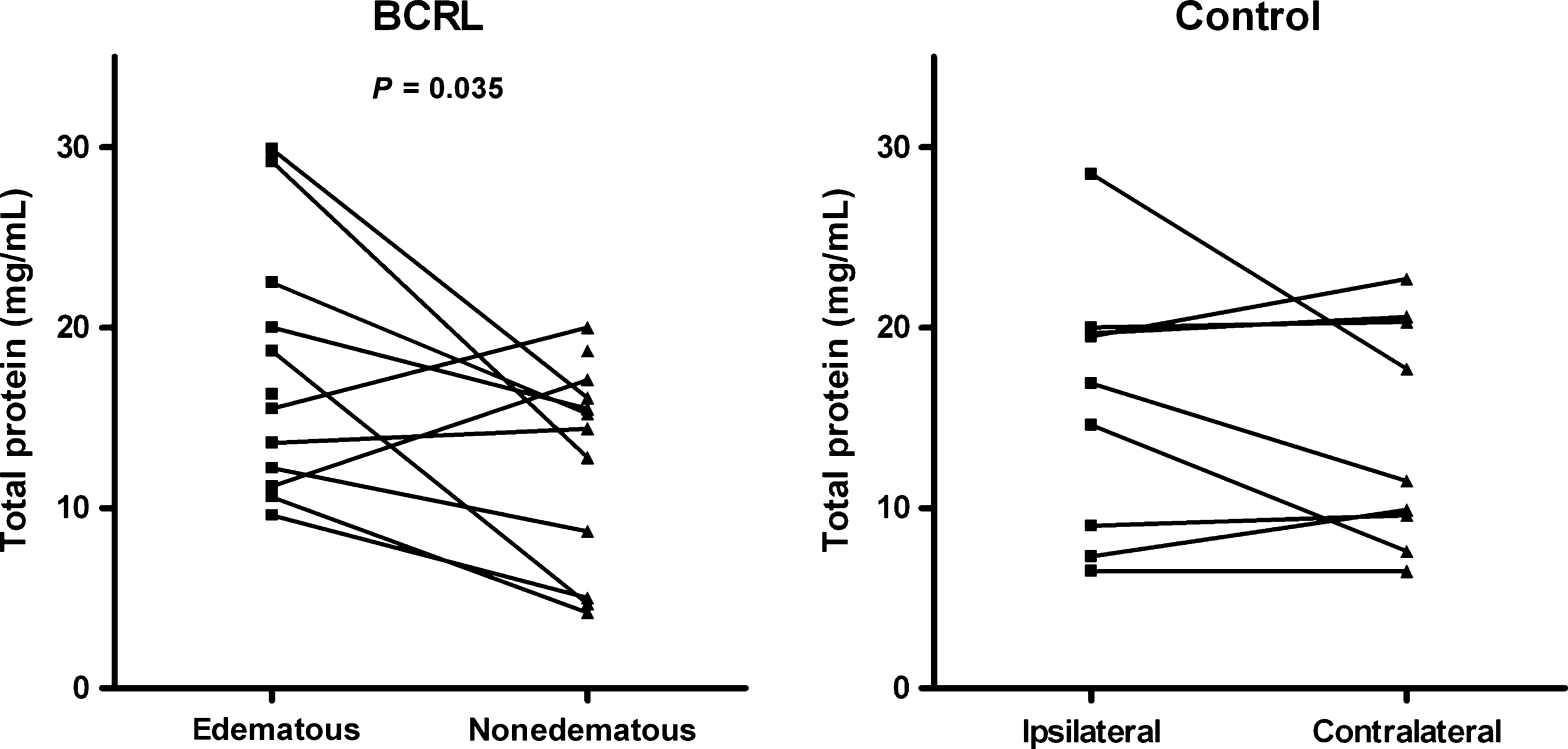
Paired measurements of total protein concentration in suction blister fluid from the edematous and nonedematous contralateral arm in the BCRL group (*n* = 11) and the ipsilateral and the contralateral arm in the control group (*n* = 9).

In the BCRL group, concentrations could not be measured in four suction blister samples from three subjects due to insufficient sample volume. Thus, paired samples from 11 subjects with BCRL were available for analysis. Paired suction blister fluid total protein concentrations for both groups are illustrated in Fig.3. In the BCRL group, suction blister total protein concentration was 15.9 (9.6–29.9) mg/mL in the edematous arms compared with 14.8 (4.2–20.0) mg/mL the paired nonedematous contralateral arms (*P* = 0.035). In the control group, total protein concentrations were 16.9 (6.5–28.5) mg/mL in the ipsilateral arms and 11.5 (6.5–22.5) mg/mL in the contralateral arms with no significant difference (*P* = 0.335). As interstitial protein concentration is highly dependent on the plasma concentration (Fadnes 1975), we also normalized total protein in suction blister fluid to plasma values. In the BCRL group, the blister-plasma ratios were 0.30 (0.12–0.56) in the edematous arms compared with 0.27 (0.05–0.42) in the nonedematous contralateral arms (*P* = 0.034). In the control group, blister-plasma ratios were 0.33 (0.11–0.48) in the ipsilateral arms 0.25 (0.11–0.43) in the contralateral arms with no significant difference (*P* = 0.355).

## Discussion

The principal and novel finding of this study is the concomitant increase in plasma VEGF-C concentration and of forearm capillary filtration capacity bilaterally in subjects with BCRL compared with matched breast cancer controls without BCRL. Furthermore, we reproduced our earlier finding of increased forearm CFC in BCRL subjects (Jensen et al. 2013) in a new population; and we found that MCP-1 and total protein concentrations are increased in suction blister fluid from edematous arms compared with the nonedematous arms in BCRL subjects. These findings will be discussed below along with study strengths, weaknesses and perspectives.

Although the cross-sectional study design does not allow us to assess causality, we find it plausible that the systemically increased levels of VEGF-C contribute to the bilateral increase in CFC, as VEGF-C has been shown to augment microvascular permeability (Hillman et al. 2001). In addition, these results support - in part - the hypothesis proposed by (Bates 2010), who has suggested that reduced interstitial fluid velocity in the lymphedematous tissue stimulates local production of VEGF-C, which due to reduced lymphatic clearance acts on the microvasculature increasing permeability. However, the present findings do not support a substantial local production in the skin. Concentrations of VEGF-C in suction blisters from the BCRL subjects seems higher compared with controls with the highest median concentration in the edematous arms, but these results were limited by the remaining sample volumes and were therefore not analyzed statistically. Furthermore, concentrations of VEGF-C in blister samples are lower than those found in plasma. Thus, measured blister VEGF-C may represent spill-over from blood, which is larger in the BCRL group due to increased microvascular permeability and a steeper concentration gradient; or it may represent a modest local production. Contrary, in support of a local VEGF-C production in the lymphedema tissue, others have found increased VEGF-C expression in BCRL skin biopsies compared with biopsies from healthy skin in the same subject (Joory et al. 2003). This finding is supported by mouse tail models of secondary lymphedema; in which VEGF-C expression in the skin increases significantly as the lymphedema develops (Rutkowski et al. 2006; Zampell et al. 2012b). Another possible source of VEGF-C production in BCRL is skeletal muscle which under normal conditions shows high expression (Joory et al. 2006). Alternatively, increased plasma VEGF-C may also be *de novo*, as it has been shown in a preliminary genetic study that patients with a specific VEGF-C haplotype have increased risk of developing BCRL (Miaskowski et al. 2013). The study design does not enable elucidation of these possibilities. As this study groups were well matched higher plasma VEGF-C and forearm CFC does not seem to be an effect of breast cancer or the treatment thereof. Hence, VEGF-C signaling seems to play a central role in BCRL pathophysiology and represents a promising target for future research in the role of VEGF-C in BCRL pathophysiology, predisposition and prediction.

This study is to our knowledge the first to directly compare concentrations of a range of selected cytokines in tissue fluid from BCRL patients with matched controls. Increased MCP-1 concentration in the edematous upper extremity compared to the nonedematous in BCRL patients supports the hypothesis, that low grade inflammation is a pathophysiologic feature of BCRL. In the present data, the MCP-1 concentration was on average three fold higher in suction blister fluid compared with plasma. We therefore conclude that MCP-1 measured in blister fluid is derived from the tissue and is not due to spill-over from plasma. A recent study on healthy volunteers using comparable methods showed that the normal inflammatory reaction to blister induction peaks after about 8 h while leukocyte counts and pro-inflammatory cytokine levels (IL-1*β*, IL-6, IL-8 and TNF-*α*) are low if blister fluid is sampled immediately after the induction (Davidsson et al. 2013). We therefore propose that the cytokine levels measured with immediate aspiration represents the immune status of the tissue. The multiplex bead array assays have proven useful for simultaneous quantification of a many cytokines in small sample volumes (Elshal and McCoy 2006); however, it has been shown that the absolute concentrations and recovery fractions measured by assays from different vendors may vary significantly; and though correlations with concentrations determined by ELISA are often acceptable, absolute concentrations can vary significantly between techniques.

Few studies exist on the role of inflammation in BCRL. One study has shown significantly increased numbers of Type 2 helper (T_H_2) cells in lymphedematous skin of 13 BCRL patients (Avraham et al. 2013), and another found inflammatory infiltrates also in skin biopsies from 27 subjects with BCRL (Lin et al. 2012). A local interstitial increase in MCP-1 is in accord with these findings as MCP-1 is a chemokine to both monocytes and to T-cells (Carr et al. 1994) and is secreted by a number of immune cells including monocytes, macrophages and dendritic cells. Furthermore, mouse models support that T-cells are pivotal in secondary lymphedema pathophysiology. In these models, inhibition of T_H_2 cell differentiation by blocking of either IL-4 or IL-13 signaling attenuate lymphedema development, and IL-4 inhibition reverses tissue swelling and remodeling in established lymphedema (Avraham et al. 2013). Furthermore, inhibition of transforming growth factor beta significantly reduced lymphedema volume, tissue fibrosis, inflammation and T_H_2 cell numbers (Avraham et al. 2010). Also macrophages, monocytes and dendritic cells are present in increased numbers, both in the skin and in the subcutaneous adipose tissue in mouse secondary lymphedema (Zampell et al. 2012a; Tabibiazar et al. 2006; Avraham et al. 2010; Lin et al. 2012; Rutkowski et al. 2006). However, if T_H_2 cells play a significant role in BCRL we would have expected high levels of IL-4, IL-5, IL-6, IL-10 and/or IL-13 in suction blister fluid from the lymphedematous arms. These cytokines were present in low concentrations and in many cases below the detection limit, and thus not likely to have biologic significance.

Adipose tissue dysfunction may play a key role in maintaining chronic low-grade inflammation in BCRL. In lean subjects, adipose tissue macrophages maintain an anti-inflammatory state through IL-10 secretion. As obesity and insulin resistance develops hypertrophic adipocytes secrete MCP-1 recruiting pro-inflammatory macrophages (McArdle et al. 2013). In murine lymphedema lymphatic stasis induces adipocyte hypertrophy and adipose tissue hyperplasia due to significant up-regulation of factors driving adipocytes differentiation (Aschen et al. 2012). Furthermore, obese mice exhibit significantly impaired lymphatic transport capacity and dendritic cell migration (Weitman et al. 2013). Our group has previously shown that obese subjects exhibit decreased subcutaneous abdominal adipose tissue lymphatic drainage, and that this does not increase upon oral glucose intake, as in lean subjects (Arngrim et al. 2013). In man, adipocyte stem cells from lymphedema tissue exhibit a transcriptional profile similar to that found in abdominal adipose tissue showing that the interstitial milieu in lymphedema promotes adipogenic differentiation (Levi et al. 2013). Thus, further research into the interplay between adipose tissue metabolism and lymphatic function seems crucial to understanding lymphedema pathophysiology.

Various techniques have been applied to sample interstitial fluid as reviewed in (Wiig and Swartz 2012). As the suction blister technique is less invasive compared to the alternative techniques e.g. the wick technique, it was applied in the present experiments. However, it is well documented that all the techniques have advantages and disadvantages, and it may be discussed which gives rise to fluid samples most correctly representing the interstitial fluid composition. It has been shown that the protein content (Haaverstad et al. 1996) and colloid osmotic pressure (Rein et al. 1988) in suction blister fluid is lower than but highly correlated with that obtained by the wick technique. The same authors have on the other hand demonstrated that the suction blister technique due to its noninvasiveness is the preferred fluid sampling method in a clinical setting in patients with leg edema after arterial reconstruction surgery (Haaverstad et al. 1997). The possible dilution effect on interstitial fluid inborn in the suction blister technique may be explained by increased filtration of plasma water caused by suction-induced low/negative hydrostatic pressure in the interstitial space (Aukland et al. 1997). We find a trend towards increased total protein in blister fluid from the edematous arms compared with that from the nonedematous arms in BCRL subjects. Although this corresponds with increased levels of MCP-1 in the same suction blisters, as MCP-1 induces increased microvascular permeability (Yadav et al. 2010), the demonstrated relative differences are modest and the biological significance unclear. Our patients had in general received early compression treatment, and had a high level of treatment adherence. Thus, although edema was objectively present in all subjects, the fluid component of the excess swelling was in general limited upon participation. Other results may have been obtained, if suction blisters were induced at the site of maximum clinical evidence of BCRL. Furthermore, fluid collection may have been performed during nonsteady state with respect to interstitial fluid volume, as the pause in compression treatment was limited to 12 h for ethical reasons. However, the linear relation found between forearm capillary filtration rates and cuff pressure shows that forearm volume was in steady state during these measurements.

The main strength of this study is that a relevant control group was included, and that groups were well matched respect to age, BMI, time since surgery, radio-chemotherapy and number of lymph nodes excised. The main limitation is the high risk of spurious significance (type 1 error) due to small groups and high number of studied parameters. We have attempted to compensate for this by applying a conservative significance level.

Collectively, the present results combined with those published by others give rise to a hypothesis for the development of BCRL. High CFC - as a constitutional biological trait - predisposes to BCRL, as the increased fluid load on the interstitium results in a reduced lymphatic drainage capacity reserve. This argument is supported by (Bains et al. 2015) in which the authors found higher lymphatic drainage rates bilaterally in forearm muscle before surgery in the breast cancer patients who later developed BCRL. A systemic increase in VEGF-C caused by BCRL development induces increased microvascular permeability overloading the remaining lymphatic drainage capacity on the operated side, but not on the contralateral side where the undisrupted lymphatic drainage remains sufficient. Prospective studies are warranted to elucidate this.

## Conclusion

Forearm capillary filtration capacity is increased bilaterally in patients with BCRL compared with matched breast cancer patients, who have not developed BCRL. This is associated with a concurrent higher plasma concentration of VEGF-C, which is known to increase capillary permeability.

## Conflict of Interest

None declared.
